# High Recovery from Either Waterlogging or Drought Overrides Any Beneficial Acclimation of *Chloris gayana* Facing a Subsequent Round of Stress

**DOI:** 10.3390/plants11202699

**Published:** 2022-10-13

**Authors:** Federico P. O. Mollard, Carla E. Di Bella, María B. Loguzzo, Agustín A. Grimoldi, Gustavo G. Striker

**Affiliations:** 1 IFEVA, CONICET, Cátedra de Fisiología Vegetal, Departamento de Biología Aplicada y Alimentos, Facultad de Agronomía, Universidad de Buenos Aires, Av. San Martín 4453, Buenos Aires C1417DSE, Argentina; 2 IFEVA, CONICET, Cátedra de Forrajicultura, Facultad de Agronomía, Universidad de Buenos Aires, Av. San Martín 4453, Buenos Aires C1417DSE, Argentina; 3 Cátedra de Fisiología Vegetal, Facultad de Agronomía, Universidad de Buenos Aires, Av. San Martín 4453, Buenos Aires C1417DSE, Argentina; 4 UWA School of Agriculture and Environment, Faculty of Science, The University of Western Australia, 35 Stirling Hwy, Crawley, WA 6009, Australia

**Keywords:** flooding, waterlogging, flooding recovery, sequential stress, water stress, allometric responses, Rhodes grass, C_4_ forage grass, acclimation

## Abstract

Climate models predict that plants will face extreme fluctuations in water availability in future global change scenarios. Then, forage production will be more frequently subjected to the destabilizing pressure of sequentially occurring waterlogging and drought events. While the isolated effects of drought (D) and waterlogging (WL) are well characterized, little is known about the consequences when both stresses occur sequentially. We hypothesized that plants sequentially subjected to opposite water scenarios (D followed by WL or vice versa) are less stress tolerant than plants experiencing repetitions of the same type of water stress (i.e., D + D or WL + WL) due to contrasting acclimation and allocation to either shoots (WL) or roots (D). *Chloris gayana* (a tropical forage grass capable of tolerating either D and WL) plants were randomly assigned to nine treatments (a sequence of two stress rounds—WL or D—each followed by a recovery phase at field capacity). Relative growth rates and allometric responses were measured after each stress round and recovery period. In the first round of stress, both WL and D reduced plant RGR similarly, despite their allocation being opposite—prioritizing shoots or roots under WL and D, respectively. The high recovery displayed after either WL or D overrode any possible acclimation of the plants facing a second round of water stress. We conclude that the tolerance of *C. gayana* to sequential water stress (either for WL or D) is likely to depend more heavily on its recovery ability than on its previous adjustment to any stress scenario that may evoke memory responses. Knowledge like this could help improve forage grass breeding and the selection of cultivars for poorly drained soils subject to sequential stress events.

## 1. Introduction

Plants are exposed to a wide range of abiotic stresses throughout their lifecycle, including extreme environmental conditions such as drought and waterlogging. Over recent years, climate change has intensified heavy rainfall and high temperature events, leading to these dynamic water scenarios [1]. Even though plants must deal with sequential stresses in nature, so far, most studies have addressed adaptive plant responses to single stress events. Moreover, recovery from stress—regarding waterlogging as a single stress—is also seldom reported [2,3]. In this work, we exposed potted plants of *Chloris gayana*—a C_4_ grass extensively used as a forage crop in pastures—to sequential events of drought and waterlogging. We have also alternated the sequences of both stresses to verify the potentially differential impact of either stress occurring early or late during the vegetative phase on plant performance (i.e., stress history). The use of *C. gayana* to test our hypotheses is based on its probed capacity to tolerate waterlogging submergence [4,5,6] and drought [7,8] and its ability to show a vigorous recovery when these are applied as single stresses.

Plant performance is constrained by water stress—either excess or deficit. At the cellular level, flooded tissues experience oxygen shortages and energy crises due to the metabolic reconfiguration from aerobic respiration to fermentation and cytoplasmic acidosis—all changes that negatively impact even non-flooded organs of the plant [9,10]. During water shortages, cells principally suffer from dehydration and loss of turgor, which impairs their expansion potential [11]. Both types of stress factors can also drive stomatal closure and falls in carbon assimilation rates [11,12,13]. In this matter, abscisic acid is the main hormone involved in both flooding and drought-induced stomatal closure [10,14], and crosstalk between both stress factors involves shared passive responses (i.e., leaves wilting) and transcriptomic, proteomic, and metabolomic common tolerance mechanisms for the maintenance of cellular homeostasis [15,16]. On the other hand, plant growth and allometry are especially sensitive to both water excess and deficits [11,17,18]. Moreover, dry mass partitioning to above and below-ground organs under excess and deficits of water in the soil is often opposite. In consequence, the shoot-to-root ratio tends to be higher under waterlogging/partial submergence and lower under drought conditions. When plants experience partial submergence, usually a higher proportion of dry mass is partitioned to shoots while root growth is hampered [4,5]. This differential dry mass allocation facilitates the emergence of a higher proportion of leaves above the water level [3,19]. Conversely, when water is scarce, an increase in roots relative to shoots has been observed—mainly due to a reduction of shoot growth [20,21], and in few cases, an increase in root biomass [22,23]. Therefore, according to the above-mentioned trade-offs in biomass partitioning between compartments in contrasting hydric conditions, we hypothesized that plants subjected sequentially to opposite water scenarios (drought followed by waterlogging or waterlogging and then drought) will be less tolerant than plants experiencing a repetition of the same water stress (i.e., drought plus drought or waterlogging plus waterlogging) due to unbalanced transpiration (shoot)/water uptake (roots) [24].

Plant growth resumption after a period of stress is critical to assess its tolerance. A literature review on waterlogging impact on forage species has shown that 7 out of 10 reports lack a recovery period in their experimental setup [25,26]; therefore, the ability of plants to resume their growth after the stress is often unknown. Moreover, plant responses to sequential water stresses followed by recovery periods have been rarely been reported (see [18] for submergence stress in *Rumex palustris* and [3] in *C. gayana*), while stress responses (second round) following a recovery period have never been reported. We propose that a prior stress can positively affect the degree of tolerance to a subsequent event with the same stress factor (e.g., either drought or flooding), considering that the first stress signature may lead to more rapid plant adjustment when responding to the second event during the post-stress period (i.e., recovery).

## 2. Results

**Figure 1:**
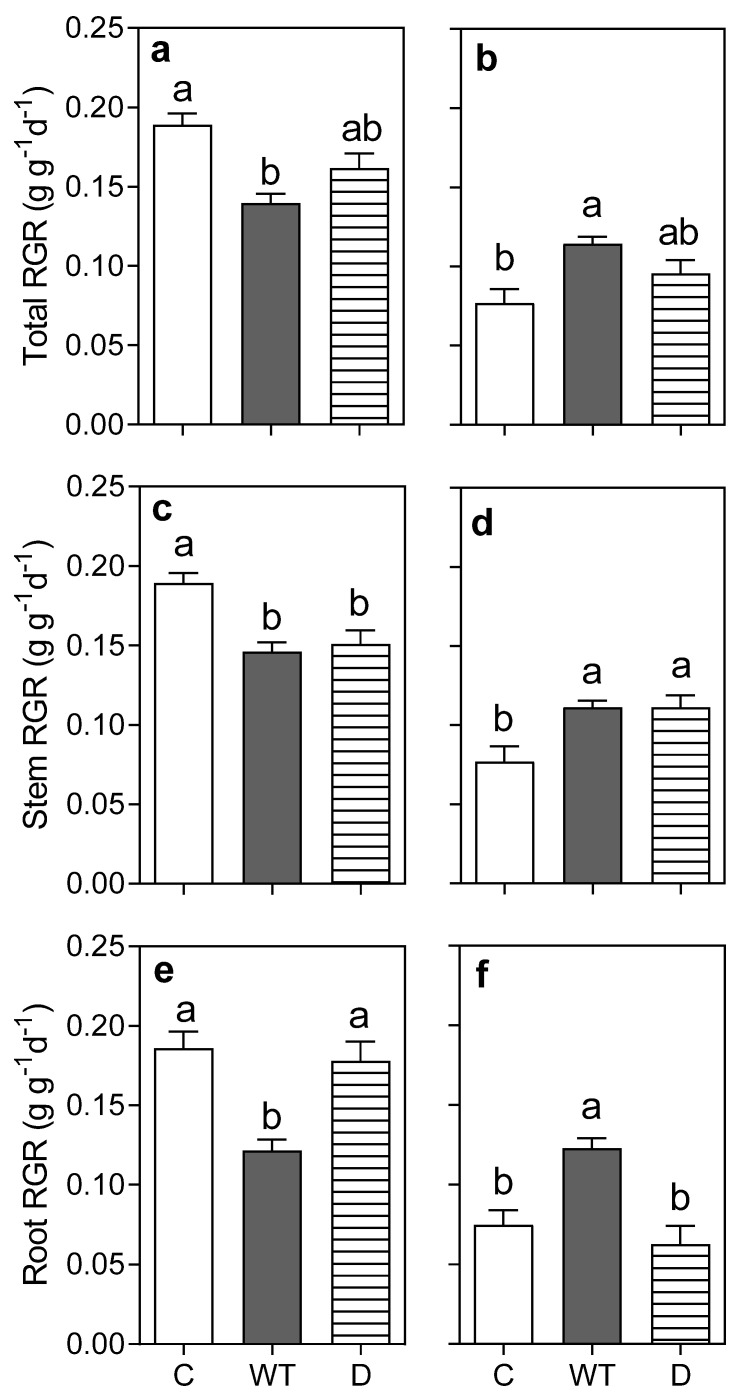
Total (**a**,**b**), shoot (**c**,**d**), and root (**e**,**f**) relative growth rates (RGR) of *Chloris gayana* plants subjected for 13 days to control conditions (C, white bars), waterlogging (WL, grey bars), or drought (D, striped bars) in the first stress round (**a**,**c**,**e**) and the subsequent 15-day recovery phase (**b**,**d**,**f**). Values are means ± e.e. (n = 8). Different letters indicate differences between treatments (*p* < 0.05).

During the first round of stress, waterlogging caused a significant effect—inhibiting the Total RGR of the *Chloris gayana* plants when compared to the control treatment (Figure 1a); however, during the first recovery phase, previously waterlogged plants overcame the control plants in terms of their Total RGR (Figure 1b; *p* < 0.05). Meanwhile, drought did not show a significant effect on Total RGR when compared to the control treatment, both during the first stress round or later during the recovery phase (Figure 1a,b; *p* > 0.05). Shoot RGR was negatively affected by both stress factors: waterlogging and drought (Figure 1c; *p* < 0.05); afterwards, the shoot RGR of the plants subjected to either stress overcame that of the control plants during the recovery phase (Figure 1d; *p* < 0.05). Figure 1e shows the idiosyncratic inhibition of root RGR caused by waterlogging (*p* < 0.05); however, during the recovery phase, previously waterlogged plants showed a significantly higher root RGR than control plants (Figure 1f; *p* < 0.05). Root RGR did not show significant differences between drought-stressed and control plants during either the first stress round or the recovery phase (Figure 1e,f; *p* > 0.05).

Plants fully recovered either total, shoot, or root biomass, and no overcompensation was observed during the recovery phase (Supplementary Material Figure S1b,d,f). Total RGR values were lower during the recovery phase than the previous 15 days (i.e., first stress round, cfr. Figure 1a vs. Figure 1b)—highlighting the effect of plant age on relative growth rate. This age condition was also recorded for shoot RGR (Figure 1c vs. Figure 1d), as well as for root RGR in control plants and plants recovering from drought (Figure 1e vs. Figure 1f); yet, previously waterlogged plants slightly increased their RGR during the recovery phase (Figure 1e,f).

**Figure 2:**
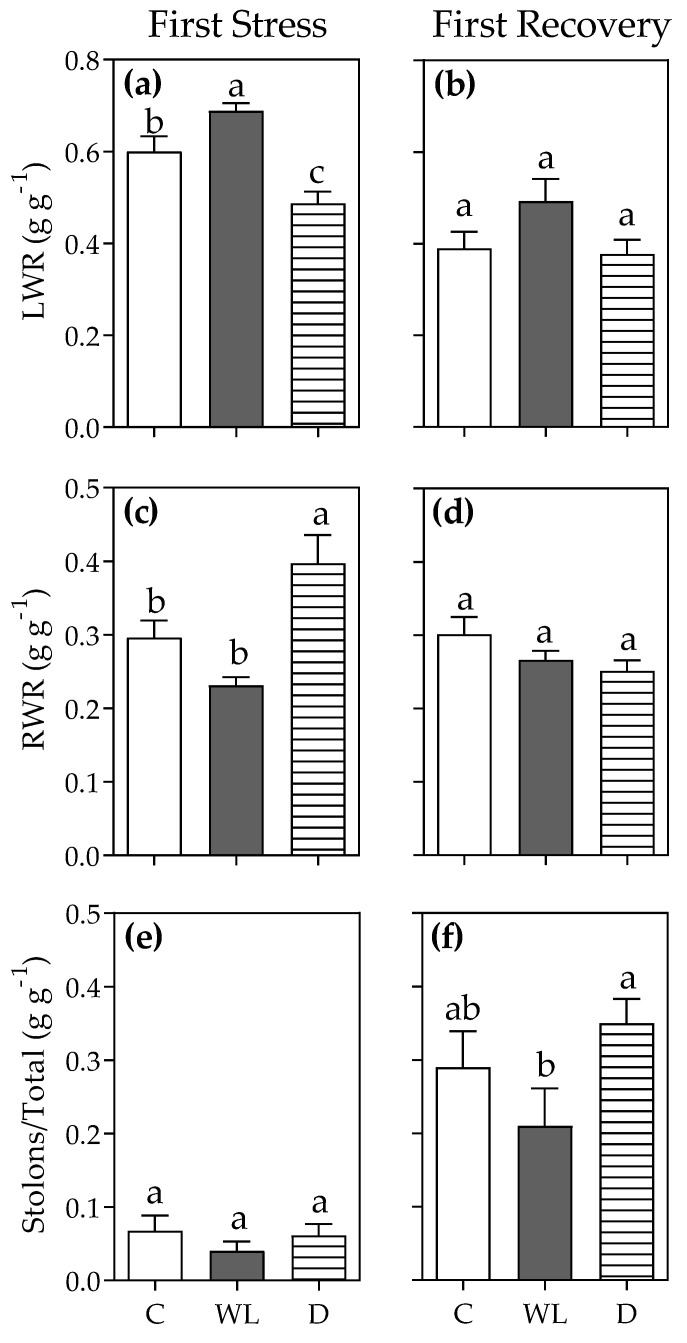
Leaf weight ratio (LWR; (**a**,**b**)), root weight ratio (RWR; (**c**,**d**)), and stolon/total ratio (**e**,**f**) of *Chloris gayana* plants subjected during 13 days to control conditions (C, white bars), waterlogging (WL, grey bars), or drought (D, striped bars) in the first stress round (**a**,**c**,**e**), and the subsequent 15-day recovery phase (**b**,**d**,**f**). Values are means ± e.e. (n = 8). Different letters indicate differences between treatments (*p* < 0.05).

A significant increase in allocation to leaves in waterlogged plants after the first stress round is shown in Figure 2a (*p* < 0.05); this promotion of LWR caused by excess water was buffered during the recovery phase (Figure 2b; *p* > 0.05). Drought-stressed plants showed a significant reduction in LWR (Figure 2a; *p* < 0.05), joined by an increase in RWR (Figure 2c; *p* < 0.05) compared to control plants. Both effects disappeared during the recovery phase (Figure 2b,d; *p* > 0.05). Stolon development was low during the first stress round in relatively young plants, and yet increased during the recovery phase (Figure 2e,f); however, after both the stress and recovery periods, waterlogged or drought plants did not significantly differ from controls (Figure 2e,f; *p* > 0.05).

**Figure 3:**
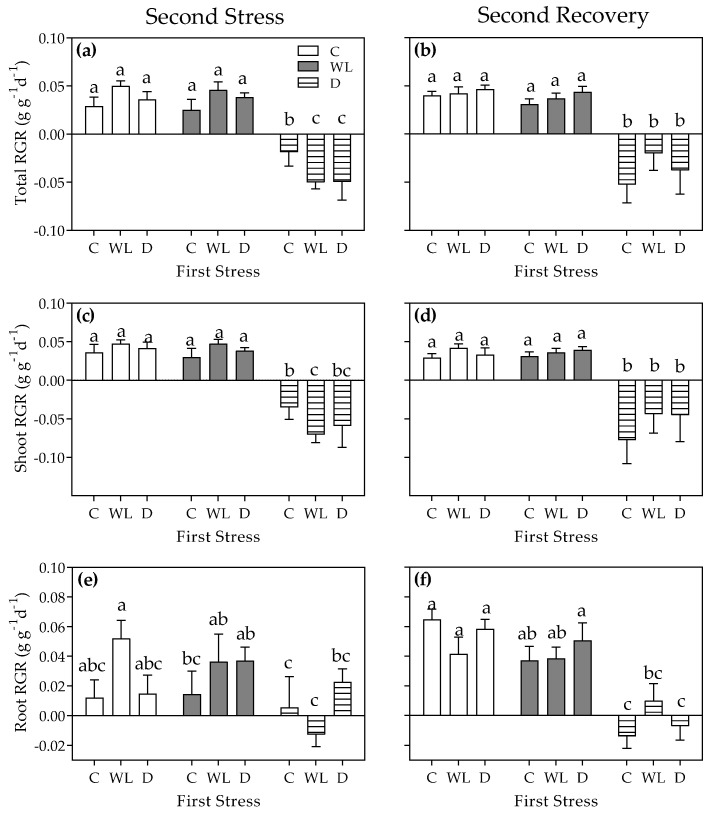
Total (**a**,**b**), shoot (**c**,**d**), and root (**e**,**f**) relative growth rates (RGR) of *Chloris gayana* plants subjected for 8 days to control conditions (C, white bars), waterlogging (WL, grey bars), or drought (D, striped bars) in the second stress round (**a**,**c**,**e**) and their subsequent 15-day recovery phase (**b**,**d**,**f**). Values are means ± e.e. (n = 8). Different letters indicate differences between treatments (*p* < 0.05).

Contrary to the expected plant hardening hypothesis, stress history did not change stress tolerance during the second stress round, as both the interaction and the first stress round were not statistically significant—as revealed by the two-way ANOVA of the RGR calculations (Figure 3a,c,e; *p* > 0.05). Accordingly, the second stress factor was statistically significant for total, shoot, and root RGRs because drought plants showed lower values than control and waterlogged plants (Figure 3a,c,e; *p* < 0.05). Noticeably, waterlogged plants did not show lower RGR values than control plants during the second stress round (Figure 3a,c,e; *p* > 0.05). RGR values during the recovery phase resembled those during the second stress round, with both the interaction and the first stress factor being non-significant (Figure 3b,d,f; *p* > 0.05). The second stress factor had a statistically significant effect during the second recovery phase, as plants subjected to drought stress did not recover and even continued to show decreasing RGR values (Figure 3b,d,f; *p* < 0.05).

**Figure 4:**
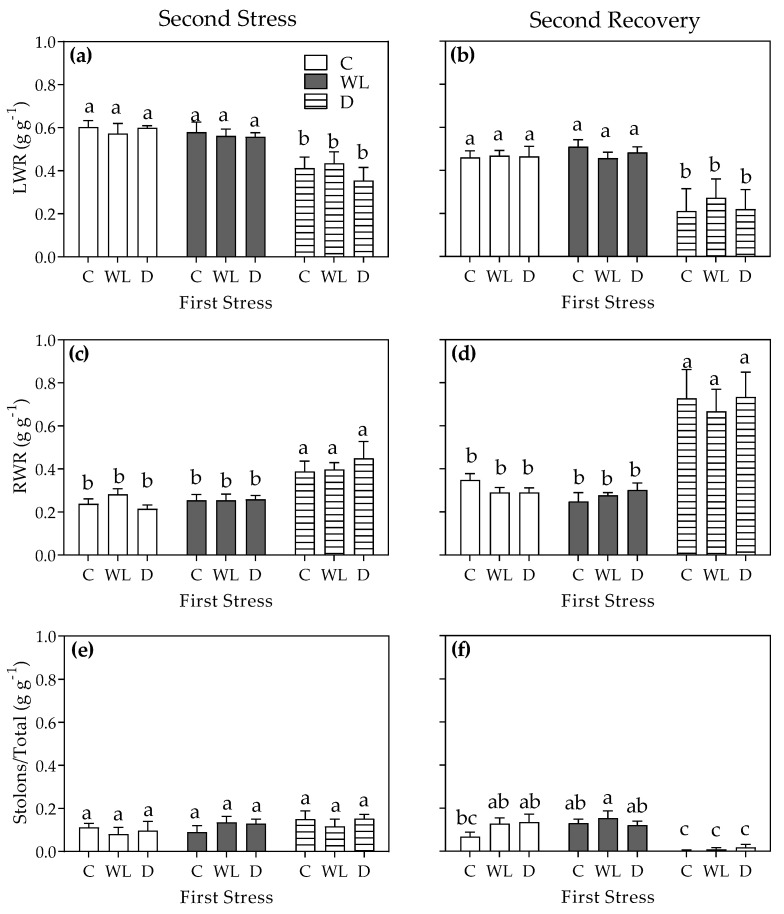
Leaf weight ratio (LWR; (**a**,**b**)), root weight ratio (RWR; (**c**,**d**)), and stolon/total ratio (**e**,**f**) of *Chloris gayana* plants subjected for 8 days to control conditions (C, white bars), waterlogging (WL, grey bars), or drought (D, striped bars) in the second stress round (**a**,**c**,**e**) and the subsequent 15-day recovery phase (**b**,**d**,**f**). Values are means ± e.e. (n = 8). Different letters indicate differences between treatments (*p* < 0.05).

Allocation was only affected by the second stress round, as both the interaction and the first stress factor did not show statistical differences between treatments (Figure 4). Accordingly, LWR was lower for drought-stressed plants compared to control plants (Figure 4a; *p* < 0.05), irrespective to the previous stress history (i.e., either from control, waterlogged, or drought conditions in the first stress round). Markedly, plants during the second stress round and with different stress histories were not affected by waterlogging compared to control plants in terms of LWR or RWR (Figure 4a,c; *p* > 0.05). Drought-stressed plants (second round) presented higher RWRs yet similar partitioning to stolons compared to control plants (Figure 4c,e; *p* < 0.05 and *p* > 0.05, respectively); again, stress history did not play a significant role in these responses. Drought plants did not recover after watering during the second recovery phase as partitioning to both leaves and stolons decreased (Figure 4b,c)—thus increasing the RWR compartment relative to control plants (Figure 4d).

## 3. Discussion

Under a climate change scenario, forage plants must be able to successfully deal with contrasting and dynamic water conditions to fulfill productivity expectations. In this work, we explored—for the very first time—plant growth responses to a second round of drought or waterlogging after full recovery from a previous water stress event. We used *Chloris gayana*, a widespread forage grass that experiences sequential drought and waterlogging conditions in large areas of its native African range [4,5,7,8], to expose plants to both stress factors, given sequentially during the vegetative phase.

### 3.1. Responses to Waterlogging, Drought, and Mixed Sequential Stresses

In contrast to young *C. gayana* plants (30 days old), which showed clear growth inhibition during the first waterlogging, adult plants (58 days old)—no matter their stress history—were fully acclimated to waterlogging conditions according to their total RGR. An increase in waterlogging tolerance along the ontogeny has been observed in several forage grasses [26,27], and it could be related to the high capacity of adult grasses to generate new adventitious aerenchymatous roots able to facilitate oxygen transport to root meristems [12,26]. By contrast, young, smaller-sized plants are expected to be less tolerant to waterlogging than adult plants, in accordance with their limited carbon fixation and potential for resource acquisition (i.e., small root system) [27,28]. Interestingly, dry mass allocation was similar between waterlogged and control plants, irrespective of their stress history—implying, non-exclusively, that (i) adult plants might be metabolically pre-acclimated to waterlogging, or (ii) the high tolerance to waterlogging of this species is not related to carbon partitioning, but relies on other traits (i.e., anato-morphological responses such as aerenchyma formation, leaf lengthening, etc.). Overall, our data suggest that adult *C. gayana* plants can withstand frequent and recurrent waterlogging without major yield losses.

Flooded plants can experience leaf dehydration during stress or even after floodwaters recede, as well as in stressful conditions shared with drought and other stress factors [15,29,30]. In relation to this, it has been shown in the crop rice that de-submergence upregulates gene transcripts associated with the acclimation to dehydration [15]. A net of shared core regulatory mechanisms are conserved across different plant stresses—as revealed by recent studies in the literature [31,32,33]—and were, consequently, expected to occur in plants acclimated to either drought or waterlogging. Besides this, previous literature indicates that abiotic stress trigger responses such as accumulation of organic metabolites and expression of ROS detoxification enzymes could acclimate *C. gayana*, as well as other forage grasses, to adverse conditions [34,35]. Considering this, a tentative hypothesis is that *C. gayana* plants acclimated to either waterlogging or drought are primed for a second stressful event and perform better in terms of growth potential to mixed stresses than previously non-stressed plants (control plants at field capacity). Despite the high sensitivity of growth-related parameters to abiotic stress [11,36], our results did not support this hypothesis, as plant performance during a second stress round in terms of RGR did not depend on the occurrence of a previous acclimation to either drought or waterlogging. Cross-protection based on hormonal (mainly abscisic acid) and metabolic acclimations was observed between drought and several stress factors capable of triggering leaf dehydration, such as heat, salinity, and freezing temperatures [37,38]. In this first exploration we could not find any evidence of cross-protection inducing hardening against waterlogging and drought.

### 3.2. Is Stress Memory Noticeable in Chloris gayana at the Whole-Plant Level?

Drought memory has been a subject of recent interest at the molecular and plant cell levels, pinpointing the importance of biochemical acclimations in obtaining a faster and stronger response to a second stress [39,40,41]. Then, our stress imposition protocol represented a good opportunity to test if drought memory can scale up to the whole-plant level in a forage grass species. In the case of *C. gayana*, we did not find an enhanced response to a second drought after the rewatering phase (i.e., expected yet ineffective, smaller decrease in either total or stem RGR—or even increased root RGR), as plants that had experienced drought performed even more poorly than plants that were well-watered during the first stress round. Moreover, plants from different backgrounds (either previously waterlogged or controls) faced second drought recovery similarly. Stress memory might not be a constitutive or conserved plant trait because it involves costs—it may hinder recovery and affect potential yield and, in consequence, be considered maladaptive [40]. On the other hand, the high recovery growth during the rehydration phase might have reset plants from any potential stress memory programmed during the first instance of drought stress, or the first stress might even have been too short to trigger the memory response where it existed. Whatever the reason, the conclusion is that short-term drought stress memory affecting growth or biomass partitioning could not be established in *C. gayana* plants.

In the highly variable environments in which *C. gayana* cultivars are being introduced, such as areas with poorly drained soils in which drought and waterlogging dynamically follow each other, plant forage yield (i.e., dry matter production) depends on a great extent to both stress acclimation and fast post-stress recovery. Our data indicate that, for *C. gayana* plants, the benefit or cost of acclimation to a first stress was offset after a full recovery as any acclimatization reached during the first stress round was disabled during the subsequent recovery phase. This result prompts further questions concerning yet-unknown relationships between the level of recovery and performance against a second abiotic stress. Those questions should be addressed in future experiments to establish if stress memory and cross-protection can help stabilize forage productivity under a more dynamic weather scenario, as is predicted from climate change models.

## 4. Materials and Methods

### 4.1. Species Description

*Chloris gayana* Kunth (Rhodes grass) is a major tropical grass cultivated worldwide as one of the most important warm-season forage grasses in subtropical and tropical areas [8]. It is used for direct grazing, producing high-quality hay and silage, and is a promising species for revegetating erodible sites. *C. gayana* is a C_4_ stoloniferous and tufted, leafy perennial grass with ascending stems (0.5–1 m tall). It has been described as tolerant to salinity and drought [42], but the species has also been introduced in areas suffering from water excess causing soil waterlogging [4,43]—such as floodplain rangelands, in which excesses and deficits in soil water can dynamically follow each other [44].

### 4.2. Plant Culture and Treatments Setup

Seeds of *C. gayana* cv. Fine Cut were germinated in an incubator at 25 °C in polystyrene boxes containing absorbent white paper saturated with distilled water. After 10 days, germinated seeds were transplanted to 3 L plastic pots (five per pot) filled with thoroughly mixed topsoil from arable cropland and sand (1:1 *v*/*v*) and transferred to a glasshouse at the Faculty of Agronomy of the University of Buenos Aires, Argentina. Seedlings were subsequently thinned to one per pot and left to grow for 30 days.

**Figure 5:**
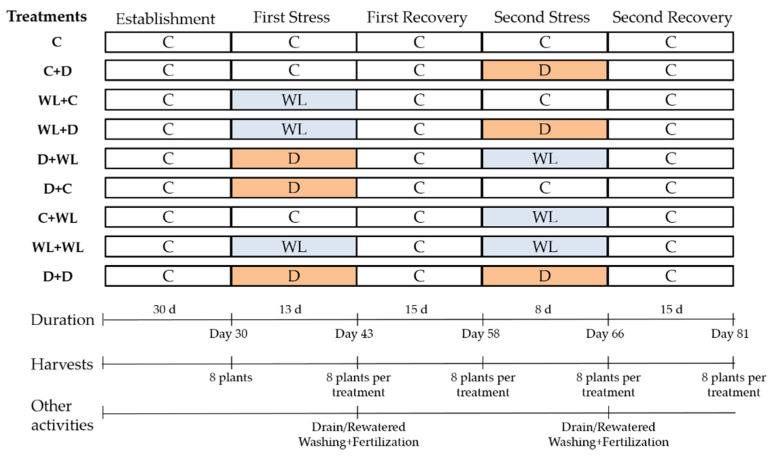
Scheme of the imposition of nine sequential stress treatments, duration of each phase (i.e., establishment, stress, and recovery), moment of harvest, and other activities performed during the experiment.

Plants with two tillers were randomly assigned to each of the nine treatments (summarized in Figure 5) and exposed to a sequence of two stress periods (waterlogging or drought), each followed by a recovery phase at field capacity. While some of the treatments mimic the seasonal transition from wetter to drier conditions during the growing season in rangelands (e.g., WL + D treatment), this experimental design not only let us subject plants to a sequence of mixed stress factors and control conditions (WL: waterlogging, D: drought, C: Control at field capacity), but also allowed us to account for plant age at the time of applying either water stress. Eight replicate plants per treatment were harvested for biomass measurements at the end of the establishment period and after each stress or recovery phase (see Figure 5).

The drought treatment guided the length of each of the stress rounds and consisted of watering withdrawal until water resupply during the recovery phase. The first stress round lasted for 13 days and finalized when 50% of the drought-stress plants had negligible stomatal conductance levels (i.e., below detection levels < 5 mmol m^−2^ s^−1^) after two consecutive clear days, as measured with a portable porometer at 10 am (Decagon Devices, Pullman, WT, USA). The second stress round lasted for 8 days and finalized when C + D plants showed symptoms of senescence in approximately 1/3 of leaves. Waterlogging (i.e., inundation water 1 cm above substrate level) was imposed by placing pots in pots of the same volume lined with impermeable plastic bags. Both recovery phases were standardized to two weeks, the length of time needed to diagnose the status of recovery after a stress period [2]. Control plants were watered every other day and drained freely. Waterlogged pots were allowed to drain at the end of each stress round, and drought plants were rewatered. Toward the end of both the establishment phase and each stress period, pots were irrigated with five times their volume to homogenize the nutrient level after each treatment. Then, pots were fertilized at a rate of 1 g per pot with 12% N, 5% P, 15% K, 2% Mg, and 8% S + micronutrients with a commercial fertilizer (DF Nitrofull S.R.L., Buenos Aires, Argentina) to prevent any potential nutrient deficits. Total evapotranspiration of drought-stressed plants was estimated as the difference between overnight rewatered pots minus their final weight (pot plus plant weight, n = 5) after each drought period. Pots were randomly arranged in the greenhouse. Mean environmental conditions in the greenhouse were 22.3 ± 5.2 °C and 63 ± 17 RH%. PAR irradiation in the greenhouse was 70% of the natural amount of sunlight. At the end of the first stress round, pots from drought-stressed plants lost on average 0.49 ± 0.02 L water through evapotranspiration. In addition, during the second stress round, pots assigned to drought-stressed plants lost on average 0.47 ± 0.03 L water, without significant differences between both drought rounds (*p* > 0.05).

### 4.3. Measurements

Dry mass accumulation per plant was measured at the end of each stress round and the recovery phase (Figure 5). After harvest, plants were dissected into each of the following parts: leaves, roots, and stolons. Stolons were included in the aerial-shoot compartment along with leaves due to its position above soil level, its importance as a propagation organ, and its role in stress tolerance (i.e., heat, drought) in some forage species [45,46]. The material was classified as senescent or alive according to a visual and tactile appraisal of plant compartments. In all cases, the material was weighed after oven drying for 72 h at 80 °C. After each of the stress rounds or recovery phases, the relative growth rates (RGRs) for each of the treatments was calculated from dry biomass data following the equation of Hunt (1982) [47]:

RGR (g/g d^−1^) = [ln (W_2_) − ln (W_1_)]/(t_2_ − t_1_) (1)

where W_2_ and W_1_ are the dry biomass at the end and the beginning of each stress or recovery period for any of the treatments (Figure 5), and t_2_ − t_1_ is the time spent in each of the periods.

Allometric relationships as Leaf Weight Ratio (LWR = Leaf weight (g)/Total weight (g)), Root Weight Ratio (RWR; RWR = Root weight (g)/Total weight (g)) and Stolon/Total (Stolon Weight (g)/Total Weight (g)) were calculated from dried biomass after each one of the harvests.

### 4.4. Statistical Analysis

One-way ANOVAs were used to determine the effect of treatments (i.e., waterlogging, drought, control) in RGRs or biomass allocation patterns after the first stress round and the first recovery phase. Interaction and treatment effects on RGRs and biomass allocation after the second stress round and recovery were evaluated by two-way ANOVAs, with ‘first stress’ and ‘second stress’ as main factors. Tukey tests at *p* < 0.05 were used to determine treatment effects.

## Data Availability

Data is contained within the article or supplementary material. Raw data is available by contacting the corresponding author.

## Supplementary Materials

The following are available online at https://www.mdpi.com/article/10.3390/plants11202699/s1, Figure S1: Total (a,b), shoot (c,d), and root (e,f) dry biomass of *Chloris gayana* plants subjected for 13 days to control conditions (C, white bars), waterlogging (WL, grey bars), or drought (D, striped bars) in the first stress round (a,c,d) and the subsequent 15-day recovery phase (b,d,f). Values are means ± e.e. (n = 8). Different letters indicate differences between treatments (*p* < 0.05). Figure S2: Total (a,b), shoot (c,d), root (e,f), and stolon (e,f) dry biomass of *Chloris gayana* plants subjected for 8 days to control conditions (C, white bars), waterlogging (WL, grey bars), or drought (D, striped bars) in the second stress round (a,c,d,e) and the subsequent 15-day recovery phase (b,d,f,g). Values are means ± e.e. (n = 8). Different letters indicate differences between treatments (*p* < 0.05).

## References

[1] Hirabayashi Y., Mahendran R., Koirala S., Konoshima L., Yamazaki D., Watanabe S., Kim H., Kanae S. (2013). Global Flood Risk under Climate Change. Nat. Clim. Change.

[2] Striker G.G. (2012). Time Is on Our Side: The Importance of Considering a Recovery Period When Assessing Flooding Tolerance in Plants. Ecol. Res..

[3] Striker G.G., Casas C., Kuang X., Grimoldi A.A. (2017). No Escape? Costs and Benefits of Leaf de-Submergence in the Pasture Grass *Chloris Gayana* under Different Flooding Regimes. Funct. Plant Biol..

[4] Imaz J.A., Giménez D.O., Grimoldi A.A., Striker G.G. (2012). The Effects of Submergence on Anatomical, Morphological and Biomass Allocation Responses of Tropical Grasses *Chloris gayana* and *Panicum coloratum* at Seedling Stage. Crop Pasture Sci..

[5] Imaz J.A., Giménez D.O., Grimoldi A.A., Striker G.G. (2015). Ability to Recover Overrides the Negative Effects of Flooding on Growth of Tropical Grasses *Chloris gayana* and *Panicum coloratum*. Crop Pasture Sci..

[6] Chiacchiera S., Bertram N., Taleisnik E., Jobbágy E. (2016). Effect of Watertable Depth and Salinity on Growth Dynamics of Rhodes Grass (*Chloris gayana*). Crop Pasture Sci..

[7] Loch D.S., Rethman N.F.G., van Niekerk W.A., Moser L., Burson B., Sollenberger L. (2004). Rhodesgrass. Warm-Season (C4) Grasses. Agronomy Monograph 45.

[8] Ponsens J., Hanson J., Schellberg J., Moeseler B.M. (2010). Characterization of Phenotypic Diversity, Yield and Response to Drought Stress in a Collection of Rhodes Grass (*Chloris gayana* Kunth) Accessions. Field Crops Res..

[9] Bailey-Serres J., Voesenek L.A.C.J. (2008). Flooding Stress: Acclimations and Genetic Diversity. Annu. Rev. Plant Biol..

[10] Yeung E., Bailey-Serres J., Sasidharan R. (2019). After The Deluge: Plant Revival Post-Flooding. Trends Plant Sci..

[11] Tardieu F., Simonneau T., Muller B. (2018). The Physiological Basis of Drought Tolerance in Crop Plants: A Scenario-Dependent Probabilistic Approach. Annu. Rev. Plant Biol..

[12] Mollard F.P.O., Striker G.G., Ploschuk E.L., Insausti P. (2010). Subtle Topographical Differences along a Floodplain Promote Different Plant Strategies among *Paspalum dilatatum* Subspecies and Populations. Austral Ecol..

[13] Di Bella C.E., Grimoldi A.A., Rossi Lopardo M.S., Escaray F.J., Ploschuk E.L., Striker G.G. (2016). Differential Growth of *Spartina densiflora* Populations under Saline Flooding Is Related to Adventitious Root Formation and Innate Root Ion Regulation. Funct. Plant Biol..

[14] Zhu J.K. (2016). Abiotic Stress Signaling and Responses in Plants. Cell.

[15] Fukao T., Yeung E., Bailey-Serres J. (2011). The Submergence Tolerance Regulator SUB1A Mediates Crosstalk between Submergence and Drought Tolerance in Rice. Plant Cell.

[16] Tamang B.G., Li S., Rajasundaram D., Lamichhane S., Fukao T. (2021). Overlapping and Stress-Specific Transcriptomic and Hormonal Responses to Flooding and Drought in Soybean. Plant J..

[17] Boyer J.S. (1970). Leaf Enlargement and Metabolic Rates in Corn, Soybean, and Sunflower at Various Leaf Water Potentials. Plant Physiol..

[18] Striker G.G., Kotula L., Colmer T.D. (2019). Tolerance to Partial and Complete Submergence in the Forage Legume Melilotus Siculus: An Evaluation of 15 Accessions for Petiole Hyponastic Response and Gas-Filled Spaces, Leaf Hydrophobicity and Gas Films, and Root Phellem. Ann. Bot..

[19] Mollard F.P.O., Striker G.G., Ploschuk E.L., Vega A.S., Insausti P. (2008). Flooding Tolerance of *Paspalum dilatatum* (Poaceae: Paniceae) from Upland and Lowland Positions in a Natural Grassland. Flora.

[20] Baruch Z. (1994). Responses to Drought and Flooding in Tropical Forage Grasses. Plant Soil.

[21] Greco S.A., Cavagnaro J.B. (2002). Effects of Drought in Biomass Production and Allocation in Three Varieties of Trichloris Crinita P. (Poaceae) a Forage Grass from the Arid Monte Region of Argentina. Plant Ecol..

[22] Hsiao T.C., Acevedo E. (1974). Plant Responses to Water Deficits, Water-Use Efficiency, and Drought Resistance. Agric. Meteorol..

[23] Hossain M.M., Liu X., Qi X., Lam H.M., Zhang J. (2014). Differences between Soybean Genotypes in Physiological Response to Sequential Soil Drying and Rewetting. Crop J..

[24] Walter J., Jentsch A., Beierkuhnlein C., Kreyling J. (2013). Ecological Stress Memory and Cross Stress Tolerance in Plants in the Face of Climate Extremes. Environ. Exp. Bot..

[25] Striker G.G., Colmer T.D. (2017). Flooding Tolerance of Forage Legumes. J. Exp. Bot..

[26] Di Bella C.E., Grimoldi A.A., Striker G.G. (2022). A Quantitative Revision of the Waterlogging Tolerance of Perennial Forage Grasses. Crop Pasture Sci..

[27] Elortegui M.D.R.I., Berone G.D., Striker G.G., Martinefsky M.J., Monterubbianesi M.G., Assuero S.G. (2020). Anatomical, Morphological and Growth Responses of *Thinopyrum Ponticum* Plants Subjected to Partial and Complete Submergence during Early Stages of Development. Funct. Plant Biol..

[28] Striker G.G., Izaguirre R.F., Manzur M.E., Grimoldi A.A. (2012). Different Strategies of *Lotus Japonicus*, *L. Corniculatus* and *L. Tenuis* to Deal with Complete Submergence at Seedling Stage. Plant Biol..

[29] Bradford K.J., Hsiao T.C. (1982). Stomatal Behavior and Water Relations of Waterlogged Tomato Plants. Plant Physiol..

[30] Setter T.L., Bhekasut P., Greenway H. (2010). Desiccation of Leaves after De-Submergence Is One Cause for Intolerance to Complete Submergence of the Rice Cultivar IR 42. Funct. Plant Biol..

[31] Ma S., Bohnert H.J. (2007). Integration of Arabidopsis Thaliana Stress-Related Transcript Profiles, Promoter Structures, and Cell-Specific Expression. Genome Biol..

[32] Kilian J., Whitehead D., Horak J., Wanke D., Weinl S., Batistic O., D’Angelo C., Bornberg-Bauer E., Kudla J., Harter K. (2007). The AtGenExpress Global Stress Expression Data Set: Protocols, Evaluation and Model Data Analysis of UV-B Light, Drought and Cold Stress Responses. Plant J..

[33] Zhang H., Sonnewald U. (2017). Differences and Commonalities of Plant Responses to Single and Combined Stresses. Plant J..

[34] Luna C., De Luca M., Taleisnik E. (2002). Physiological Causes for Decreased Productivity under High Salinity in Boma, a Tetraploid *Chloris Gayana* Cultivar. II. Oxidative Stress. Aust. J. Agric. Res..

[35] Wang X., He Y., Zhang C., Tian Y.A., Lei X., Li D., Bai S., Deng X., Lin H. (2021). Physiological and Transcriptional Responses of *Phalaris Arundinacea* under Waterlogging Conditions. J. Plant Physiol..

[36] Mollard F.P.O., Foote A.L., Wilson M.J., Crisfield V., Bayley S.E. (2013). Monitoring and Assessment of Wetland Condition Using Plant Morphologic and Physiologic Indicators. Wetlands.

[37] Sabehat A., Lurie S., Weiss D. (1998). Expression of Small Heat-Shock Proteins at Low Temperatures. A Possible Role in Protecting against Chilling Injuries. Plant Physiol..

[38] Kong R.S., Henry H.A.L. (2016). Prior Exposure to Freezing Stress Enhances the Survival and Recovery of Poa Pratensis Exposed to Severe Drought. Am. J. Bot..

[39] Bruce T.J.A., Matthes M.C., Napier J.A., Pickett J.A. (2007). Stressful “Memories” of Plants: Evidence and Possible Mechanisms. Plant Sci..

[40] Crisp P.A., Ganguly D., Eichten S.R., Borevitz J.O., Pogson B.J. (2016). Reconsidering Plant Memory: Intersections between Stress Recovery, RNA Turnover, and Epigenetics. Sci. Adv..

[41] Jacques C., Salon C., Barnard R.L., Vernoud V., Prudent M. (2021). Drought Stress Memory at the Plant Cycle Level: A Review. Plants.

[42] Dear B.S., Reed K.F.M., Craig A.D. (2008). Outcomes of the Search for New Perennial and Salt Tolerant Pasture Plants for Southern Australia. Aust. J. Exp. Agric..

[43] Boschma S.P., Lodge G.M., Harden S. (2008). Herbage Mass and Persistence of Pasture Legumes and Grasses at Two Potentially Different Saline and Waterlogging Sites in Northern New South Wales. Aust. J. Exp. Agric..

[44] Di Bella C.E., Striker G.G., Loreti J., Cosentino D.J., Grimoldi A.A. (2016). Soil Water Regime of Grassland Communities along Subtle Topographic Gradient in the Flooding Pampa (Argentina). Soil Water Res..

[45] Rutledge J.M., Volenec J.J., Hurley R.H., Reicher Z.J. (2012). Seasonal Changes in Morphology and Physiology of Roughstalk Bluegrass. Crop Sci..

[46] Boller B.C., Nösberger J. (1983). Effects of Temperature and Photoperiod on Stolon Characteristics, Dry Matter Partitioning, and Nonstructural Carbohydrate Concentration of Two White Clover Ecotypes. Crop Sci..

[47] Hunt R. (1982). Plant Growth Analysis: Second Derivatives and Compounded Second Derivatives of Splined Plant Growth Curves. Ann. Bot..

