# Supplementary material for: High Recovery from Either Waterlogging or Drought Overrides Any Beneficial Acclimation of Chloris gayana Facing a Subsequent Round of Stress
Source: Plants (Basel). 2022 Oct 13;11(20):2699. doi: 10.3390/plants11202699 (PMC9610420; doi:10.3390/plants11202699)
Supplement: Supplementary file 1 [file plants-11-02699-s001.zip › plants-1892561-supplementary.pdf]

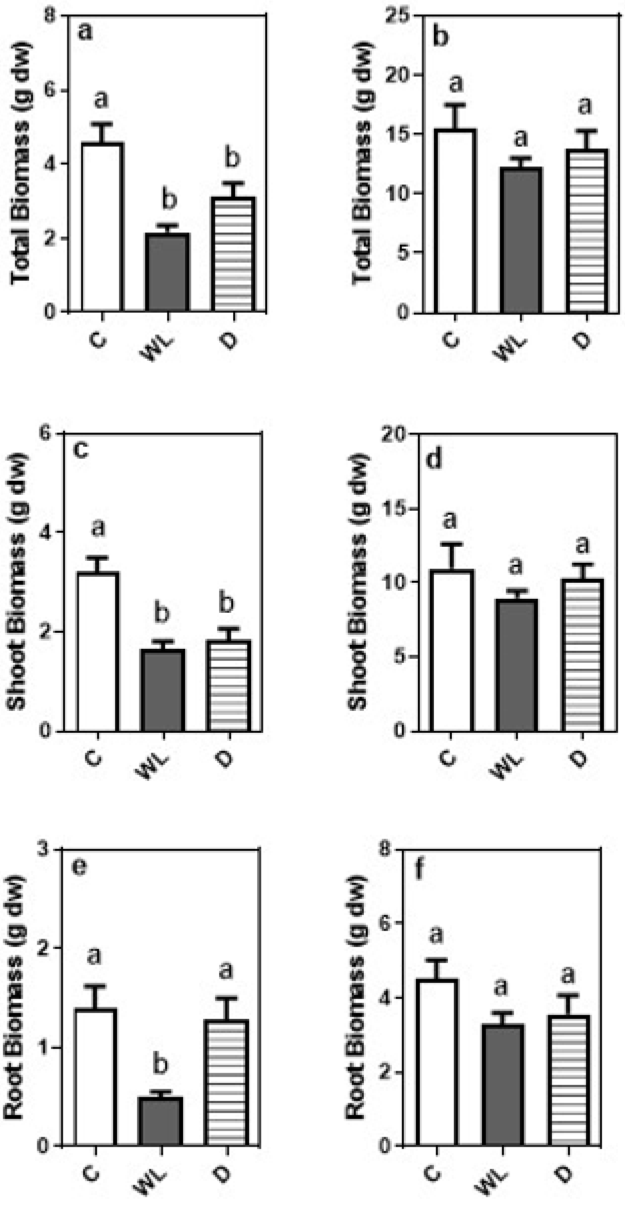

Figure S1: Total (a,b), shoot (c,d), and root (e,f) dry biomass of Chloris gayana plants subjected for 13 days to control conditions (C, white bars), waterlogging (WL, grey bars), or drought (D, striped bars) in the first stress round (a,c,d) and the subsequent 15-day recovery phase (b,d,f). Values are means ± e.e. (n = 8). Different letters indicate differences between treatments ($p < 0.05$).

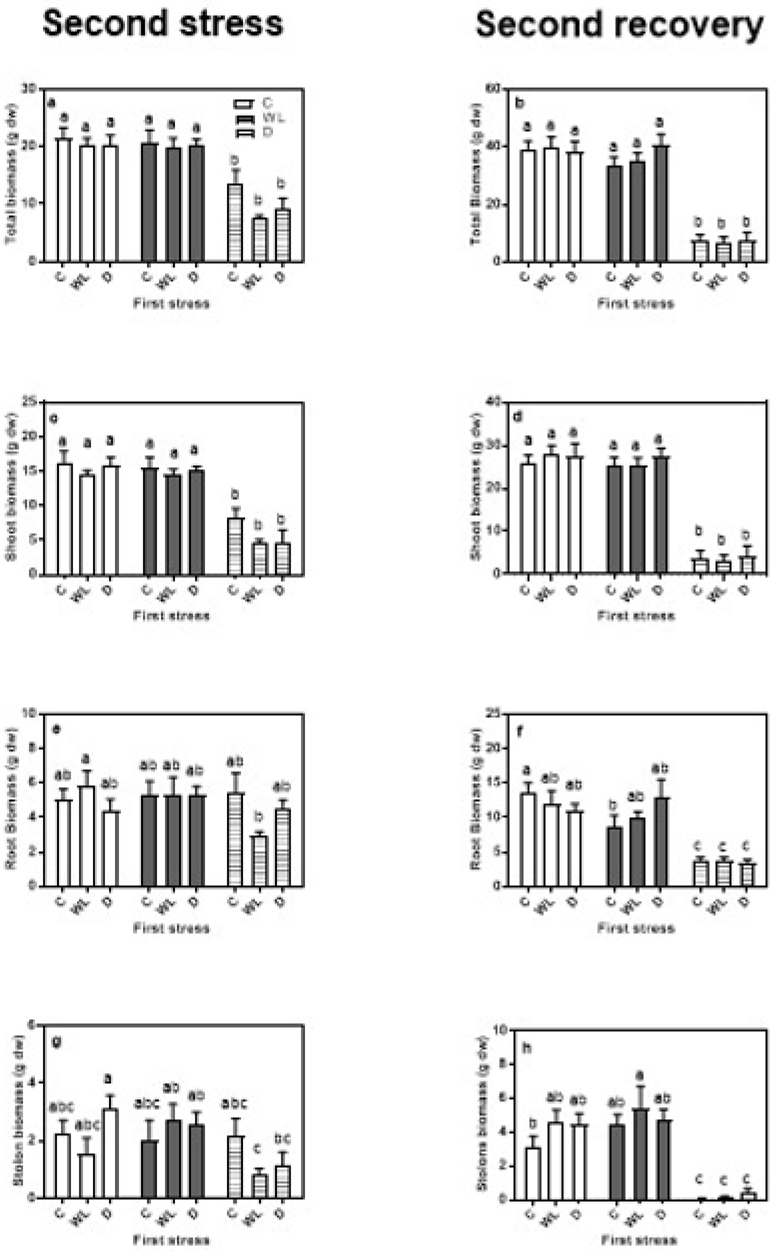

Figure S2: Total (a,b), shoot (c,d), root (e,f), and stolon (e,f) dry biomass of Chloris gayana plants subjected for 8 days to control conditions (C, white bars), waterlogging (WL, grey bars), or drought (D, striped bars) in the second stress round (a,c,d,e) and the subsequent 15-day recovery phase (b,d,f,g). Values are means ± e.e. (n = 8). Different letters indicate differences between treatments ($p < 0.05$).
